# Effect of Rifampin on Thyroid Function Test in Patients on Levothyroxine Medication

**DOI:** 10.1371/journal.pone.0169775

**Published:** 2017-01-12

**Authors:** Hye In Kim, Tae Hyuk Kim, Hosu Kim, Young Nam Kim, Hye Won Jang, Jae Hoon Chung, Seong Mi Moon, Byung Woo Jhun, Hyun Lee, Won-Jung Koh, Sun Wook Kim

**Affiliations:** 1 Division of Endocrinology & Metabolism, Department of Medicine, Thyroid Center, Samsung Medical Center, Sungkyunkwan University School of Medicine, Seoul, South Korea; 2 Department of Medical Education, Sungkyunkwan University School of Medicine, Seoul, South Korea; 3 Division of Pulmonary and Critical Care Medicine, Department of Medicine, Samsung Medical Center, Sungkyunkwan University School of Medicine, Seoul, South Korea; University of Rochester, UNITED STATES

## Abstract

**Background:**

Levothyroxine (LT4) and rifampin (RIF) are sometimes used together; however, no clinical studies have assessed the effects of these drugs on thyroid function or the need to adjust LT4 dose.

**Methods:**

We retrospectively reviewed the records of 71 Korean patients who started RIF during LT4 treatment. Clinically relevant cases that required dose adjustment according to the American Thyroid Association (ATA)/American Association of Clinical Endocrinologists (AACE) guidelines were identified, and risk factors of increased LT4 dose were analyzed.

**Results:**

After administering RIF, median serum thyroid-stimulating hormone (TSH) level (2.58 mIU/L, interquartile range [IQR] 0.21–7.44) was significantly higher than that before RIF (0.25 mIU/L, IQR, 0.03–2.62; P < 0.001). An increased LT4 dose was required for 50% of patients in the TSH suppression group for thyroid cancer and 26% of patients in the replacement group for hypothyroidism. Risk factor analysis showed that remaining thyroid gland (odds ratio [OR] 9.207, P = 0.002), the time interval between starting RIF and TSH measurement (OR 1.043, P = 0.019), and baseline LT4 dose per kg body weight (OR 0.364, P = 0.011) were clinically relevant variables.

**Conclusions:**

In patients receiving LT4, serum thyroid function test should be performed after starting RIF treatment. For patients with no remnant thyroid gland and those receiving a lower LT4 dose, close observation is needed when starting RIF and TB medication.

## Introduction

Levothyroxine (LT4) is widely used to replace thyroid hormone in patients with hypothyroidism and to suppress serum thyroid-stimulating hormone (TSH) for the treatment of differentiated thyroid cancer (DTC). The primary pathway of LT4 metabolism is sequential deiodination. The main site of deiodination is the liver, although several other organs including the kidneys also metabolize LT4. LT4 is a substrate for CYP3A4, a member of the hepatic cytochrome P450 family of enzymes. Approximately 80% of LT4 is eliminated by the kidneys, and the remaining 20% is eliminated in the stool [1].

Rifampin (RIF) is used to treat active tuberculosis (TB) and latent TB infection (LTBI), as well as disease caused by nontuberculous mycobacteria (NTM) such as *Mycobacterium avium* complex. These mycobacterial diseases require long-term RIF treatment. RIF is a potent inducer of hepatic cytochrome P450 enzymes, especially CYP3A4, thus increasing the metabolism of many drugs that are metabolized by the liver [2,3].

In some patients undergoing LT4 treatment, elevated TSH leads to an increase in LT4 dose after administration of RIF. The American Thyroid Association (ATA)/American Association of Clinical Endocrinologists (AACE) hypothyroidism guidelines recommend measuring TSH within 4–8 weeks after starting a drug that might influence LT4 dose, such as RIF [4]. However, only a few case reports have noted LT4 and RIF drug interactions leading to elevated TSH [5–7]. Furthermore, no retrospective or prospective clinical studies in the English language literature have evaluated elevated TSH level after administering RIF in patients receiving LT4.

In this study, we investigated the effect of RIF on thyroid function in patients undergoing LT4 therapy. We also analyzed risk factors of a clinically relevant change in thyroid function.

## Materials and Methods

### Study population

This study was a retrospective cohort study of adults who started RIF treatment during LT4 treatment between November 1994 and June 2014 at Samsung Medical Center (a 1,961-bed referral hospital in Seoul, South Korea). Patients 18 years or younger and those who took RIF for diseases other than TB, LTBI, or NTM infection were not included. We also excluded patients administered RIF for less than 4 weeks and patients with an obvious cause for a change in thyroid function, including LT4 discontinuation due to poor compliance or in preparation for radioiodine treatment. Seventy-one Korean patients were enrolled in this study. The study population was divided into two groups based on the reason for LT4 treatment: LT4 replacement to manage hypothyroidism and TSH suppression to manage DTC.

### Study design and outcome measurement

The primary outcome of this study was change in thyroid function as measured by serum TSH and free thyroxine (fT4) after RIF administration. The secondary outcome was the percentage of patients with a clinically relevant change in thyroid function leading to a need for LT4 dose adjustment. A clinically relevant change in thyroid function was defined as a change that required LT4 dose adjustment according to the ATA/AACE guidelines. In the LT4 replacement group, a serum TSH level that exceeded the upper normal limit after starting RIF was classified as clinically relevant by 2012 ATA/AACE hypothyroidism guidelines [4]. For the suppression group, a clinically relevant change was defined as a serum TSH increase above the target TSH range according to the revised 2015 ATA guidelines for DTC after RIF administration [8,9].

The following factors were investigated for possible biological and/or pharmacological associations with LT4 dose adjustment: age, sex, reason for LT4 treatment, LT4 dose, reason for RIF treatment, RIF dose, time interval between starting RIF and serum TSH measurement, underlying liver disease, and underlying renal disease [10,11].

### Statistical analysis

All variables including baseline characteristics are presented as number with percentage for categorical variables, mean ± standard deviation (SD) for continuous variables following a normal distribution, and median with interquartile range (IQR) for continuous variables not following a normal distribution. Changes in serum TSH and serum free T4 were evaluated using the Wilcoxon signed rank test (Wilcoxon matched pairs test). Bivariate analysis of variables between clinically relevant and clinically irrelevant changes in thyroid function were performed with the Student’s t-test for continuous variables following a normal distribution, Mann-Whitney *U* test for continuous variables not following a normal distribution, Chi-square test for categorical variables, and Fisher’s exact test for categorical variables with an expected frequency less than five. Age, sex, liver disease, renal disease, RIF-TSH duration, remnant thyroid, etiology of RIF, RIF dose, and LT4 dose were used as covariates of multivariable analysis. Binary logistic regression with likelihood ratio test and backward elimination was used for multivariable analysis. All statistical analyses were performed with SPSS Statistical Software package version 22.

### Ethics

The data used in this study were made available in an anonymized format, and the need for informed consent was waived. This retrospective observational study was approved by the Institutional Review Board of Samsung Medical Center (IRB No. 2015-08-101), and full permission was granted to review and publish information obtained from patient records.

## Results

The study population (N = 71) was divided into a TSH suppression group who received LT4 to suppress DTC recurrence (N = 46) and a TSH replacement group who took LT4 to replace thyroid hormone (N = 25). Baseline characteristics of the study population are presented in Table 1. Most patients were female (N = 58, 82%) and the median age was 52 years (IQR, 46–60 years). Median LT4 dose was 2.00 μg/kg/day (IQR, 1.49–2.54). The suppression group had a higher LT4 dose than did the replacement group. The most common reason to administer RIF was TB (N = 49, 69%). The median time between the start of RIF and serum TSH measurement was 32 weeks (IQR, 20–42 weeks). Baseline serum TSH level before administering RIF was 0.25 mIU/L (median, IQR, 0.03–2.62 mIU/L), and baseline serum fT4 level was 1.34 ng/dL (median, IQR, 1.12–1.61 ng/dL). Some patients had underlying liver disease (N = 11, 15%) or kidney disease (N = 5, 7%).

**Table 1:** Baseline characteristics of enrolled patients taking levothyroxine before starting rifampin.

	TSH suppression group (*n* = 46)	LT4 replacement group (*n* = 25)	Total (*n* = 71)
Age, median (IQR)	49 (43–59)	57 (50–66)	52 (46–60)
Female, n (%)	40 (87)	18 (72)	58 (82)
Body weight, kg, median (IQR)	56 (51–61)	55 (51–66)	56 (51–62)
LT4 dose, μg/kg/day, median (IQR)	2.15 (1.83–3.13)	1.47 (0.98–2.34)	2.00 (1.49–2.54)
Reason for RIF administration			
TB, n (%)	28 (60)	18 (72)	49 (69)
LTBI, n (%)	3 (6)	2 (8)	5 (7)
NTM, n (%)	15 (32)	5 (20)	17 (23)
RIF dose, mg/kg/week, median (IQR)	70 (55–77)	64 (54–73)	69 (54–76)
Time after starting RIF, weeks, median (IQR)	37 (22–43)	25 (15–38)	32 (20–42)
Baseline TSH level, mIU/L, median (IQR)	0.16 (0.01–1.39)	2.30 (0.19–9.85)	0.25 (0.03–2.62)
Baseline fT4 level*, ng/dL, median (IQR)	1.53 (1.29–1.65)	1.20 (0.88–1.29)	1.34 (1.12–1.61)
Underlying liver disease, n (%)	2 (4)	9 (36)	11 (15)
Underlying kidney disease, n (%)	3 (6)	2 (8)	5 (7)

*For 27 patients and 17 patients in the TSH suppression and LT4 replacement groups, respectively.

All categorical variables are expressed as a percentage (%) and all continuous values are expressed as the median and interquartile range (IQR) because none of the variables follow a standard distribution.

TSH, thyroid-stimulating hormone; LT4, levothyroxine; RIF, rifampin; TB, active tuberculosis; LTBI, latent TB infection; NTM, nontuberculous mycobacteria disease; fT4, free thyroxine.

Table 2 shows serum TSH and fT4 levels before and after administering RIF. Median serum TSH levels before and after administering RIF were 0.25 mIU/L (IQR, 0.03–2.62 mIU/L) and 2.58 mIU/L (IQR, 0.21–7.44 mIU/L), respectively. Serum TSH levels after administering RIF were significantly higher than those before administering RIF (P < 0.001). Conversely, median serum fT4 levels decreased significantly after RIF was administered (1.34 ng/dL [IQR 1.12–1.61] to 1.01 [0.90–1.28], P < 0.001). Fifty percent of patients in the TSH-suppression group (23/46) and 24% in the simple replacement group (6/25) experienced changes in TSH that required an increase in LT4 dose according to ATA/AACE guidelines.

**Table 2:** Thyroid profile before and after RIF administration.

	Before RIF	After RIF	P-value
**TSH, mIU/L, median(IQR)**	0.25 (0.03–2.62)	2.58 (0.21–7.44)	P < 0.001^a^
**fT4, ng/dL, median(IQR)**	1.34 (1.12–1.61)	1.01 (0.90–1.28)	P < 0.001^a^

^a^Wilcoxon signed rank test (Wilcoxon matched pairs test)

Abbreviations: TSH, thyroid-stimulating hormone; RIF, rifampin; fT4, free thyroxine

When patients with clinically relevant and irrelevant changes were compared, remnant thyroid (P = 0.002, Chi-square test), reason for LT4 treatment (P = 0.04, Chi-square test), and time between starting RIF and TSH measurement (P = 0.005, Mann-Whitney *U* test) were significantly different (Table 3). Other variables such as age, sex, LT4 dose, reason for administering RIF, RIF dose, underlying liver disease, and underlying renal disease did not differ significantly between groups. In multivariate analysis, lack of remnant thyroid (hazard ratio [OR] 9.20, 95% confidence interval [CI] 2.28–37.17, P = 0.002), time between starting RIF and serum TSH measurement (OR 1.04, 95% CI 1.00–1.08, P = 0.019), and LT4 dose per kg body weight (OR 0.36, 95% CI 0.16–0.79, P = 0.011) were clinically significant (Table 4, Model 2). More detailed statistical values for each statistics in Tables 2–4 could be found in S1 Table. For every one-week increase in the time between the start of RIF and TSH measurement, the odds of an LT4 dose increased 1.04 times. However, the odds were no longer clinically significant beyond 40 weeks after the start of RIF when logistic regression was performed stepwise according to time interval.

**Table 3:** Risk factor analysis (bivariate) of clinically relevant changes in TSH.

Variables		Clinically non-relevant (n = 42)	Clinically relevant (n = 29)	P-value
Age, median (IQR)		54 (45–60)	49 (46–65)	0.497 ^b^
Male, n (%)		8 (19)	5 (17)	0.847 ^a^
Reason for LT4, n (%)	PTC	23 (54)	23 (79)	**0.04** ^a^
	Others	19 (45)	6 (20)	
Reason for RIF, n (%)	TB	31 (73)	18 (62)	0.503 ^a^
	LTBI	2 (4)	3 (10)	
	NTM	9 (21)	8 (27)	
Remnant thyroid, n (%)		30 (71)	10 (34)	**0.002** ^a^
LT4 dose, μg/kg/day, mean ± SD		2.20 ± 1.12	2.04 ± 0.63	0.434 ^b^
RIF dose, mg/kg/week, median (IQR)		69 (55–74)	70 (45–77)	0.874 ^b^
Time after starting RIF, weeks, median (IQR)		26 (15–38)	41 (23–57)	**0.005** ^b^
Baseline TSH level, mIU/L, Median (IQR)		0.21 (0.10–4.62)	0.29 (0.08–2.40)	0.906 ^b^
Baseline fT4 level, ng/dL, mean ± SD		1.30 ± 0.09	1.42 ± 0.06	0.349 ^b^
Underlying liver disease, n (%)		8 (19)	6 (20)	0.864 ^a^
Underlying kidney disease, n (%)		3 (7)	2 (6)	1.000 ^a^

^a^Chi-square test

^b^Mann-Whitney *U* test

TSH, thyroid-stimulating hormone; LT4, levothyroxine; RIF, rifampin; TB, active tuberculosis; LTBI, latent TB infection; NTM, nontuberculous mycobacteria disease; fT4, free thyroxine; PTC, papillary thyroid cancer

**Table 4:** Risk factor analysis (multivariate) of clinically relevant cases.

	Variable	OR (95% CI)	P-value
**Model 1**	No remnant thyroid	3.52 (1.21–10.26)	0.021 ^a^
	Time after starting RIF	1.03 (1.00–1.06)	0.034 ^a^
**Model 2**	No remnant thyroid	9.20 (2.28–37.17)	0.002^a^
	Time after starting RIF	1.04 (1.00–1.08)	0.019^a^
	LT4 dose	0.36 (0.16–0.79)	0.011^a^

^a^Binary logistic regression analysis with backward LRT

Model 1: age, sex, RIF-TSH duration, remnant thyroid

Model 2: Model 1 + liver disease, renal disease, etiology of RIF, RIF dose, LT4 dose

‘Reason for LT4 treatment’ was not included in the logistic regression model because of multi-collinearity with ‘remnant thyroid.’

TSH, thyroid-stimulating hormone; LT4, levothyroxine; RIF, rifampin

## Discussion

In this retrospective analysis, we investigated the effects of RIF on the thyroid function of patients who were taking LT4. Our data showed that serum TSH level significantly increased and serum fT4 level significantly decreased after starting RIF treatment. Furthermore, 50% of patients in the TSH-suppression group and 26% of patients in the replacement group required an LT4 dose increase to maintain target serum TSH according to the ATA/AACE guidelines. Our results support recommendation 16 of the ATA/AACE hypothyroidism guidelines, which recommends measuring TSH within 4–8 weeks after starting a drug, such as RIF, that might influence LT4 dose [4].

In contrast, Christensen et al. reported no significant change in serum TSH or fT4 in 13 healthy volunteers who took RIF for 28 days [12]. In their study, thyroid volume and antipyrine clearance increased significantly, but there were no significant changes in serum TSH and fT4 levels. This result implies that RIF increased T4 clearance in the liver, but did not change serum TSH or fT4 due to compensation by the normal thyroid gland. This discrepancy likely reflects differences in study populations. Patients who take LT4 in normal clinical practice usually have little or no remaining thyroid function to compensate for increases in LT4 clearance. In fact, a lack of remaining thyroid and lower LT4 dose upon starting RIF were risk factors of clinically relevant changes in thyroid function in our study.

LT4 dose per kg body weight at the time of RIF administration was a significant risk factor in multivariate analysis, but not in bivariate analysis. This result could be explained by the confounding effect of remaining thyroid gland. The clinically irrelevant group contained more patients with remaining thyroid who were taking lower doses of LT4 and patients who were already taking a relatively high dose of LT4 before starting RIF compared with the clinically relevant group. Use of the mean LT4 dose in bivariate analysis might have masked any differences in LT4 dose. However, when we adjusted for remaining thyroid (see methods), LT4 dose per kg body weight became a significant risk factor.

The time between starting RIF and measuring serum TSH was a risk factor of LT4 dose adjustment in both bivariate and multivariate analyses; however, this effect disappeared 40 weeks after starting RIF. RIF induces CYP3A4 in approximately 3 days. However, when another drug (i.e., a CYP3A4 substrate) is added, the new equilibrium depends on the second drug, an effect known as drug-drug interaction (DDI). For example, a combination of steroid and RIF requires 2 weeks to reach a new equilibrium. Dynamic DDI modeling with RIF revealed that the new equilibrium time is proportional to the half-life of the substrate [10,13]. The half-life of prednisolone is 12–36 hours and reaches a new equilibrium with RIF in about 2 weeks. The exact equilibrium time of RIF and LT4 has not been determined, but more than 4–8 weeks might be required to reach a new equilibrium because the half-life of LT4 (approximately 190 hours) is much longer than that of prednisolone. Our data suggest that TSH level should be checked periodically at 4–8 weeks as recommended by 2012 ATA/AACE hypothyroidism guidelines, but also at about 40 weeks.

Past studies have documented that isoniazide (INH) is the substrate of N-acetyltransferase. The slow-acetylator phenotype (poor metabolizers) is prone to INH side effects due to increased blood INH concentration. INH inhibits CYP3A, contrary to RIF, and thus RIF might have a weaker effect on CYP3A in slow acetylators. In this study, most patients took INH and RIF together, but all patients were Korean and only 10–15% of Koreans are expected to be slow acetylators [14–16]. In contrast, in Caucasians and African Americans, the prevalence of slow acetylators is approximately 50% [17–19]. Considering that the prevalence of slow acetylators is only 10–15% in Koreans while there are 50% in Caucasians and African Americans, the effect of RIF with con-concomitant INH on LT4 clearance might have been more prominent in this study population than other ethnicities.

The small study population from a single tertiary referral center is a limitation of this study, and a further study in a larger population is warranted. In addition, quality of life (for example, symptoms of hypothyroidism such as general weakness or weight gain) was not investigated because of the weakness of the retrospective design.

In conclusion, serum TSH increased and fT4 decreased significantly after the start of RIF treatment in patients taking LT4 for either hormone replacement for hypothyroidism or TSH suppression to manage DTC. Therefore, clinicians should closely monitor thyroid function when RIF is administered, particularly in patients without remaining thyroid gland and those taking a low dose of LT4. Further studies are needed to confirm these results, especially in populations where slow acetylators are more prevalent, such as in Caucasians and African Americans.

## Supporting Information

S1 Table (DOCX)
