# Supplementary material for: Effect of Rifampin on Thyroid Function Test in Patients on Levothyroxine Medication
Source: PLoS One. 2017 Jan 12;12(1):e0169775. doi: 10.1371/journal.pone.0169775 (PMC5231266; doi:10.1371/journal.pone.0169775)
Supplement: S1 Table — (DOCX) [file pone.0169775.s001.docx]

**S1 Table.** Statistical values of each statistical test

| **Wilcoxon matched paired test (Table 2)** | | | | | |
| --- | --- | --- | --- | --- | --- |
| Variables | | Before RIF | After RIF | P-value | **Z-value*** |
| TSH, mIU/L, median(IQR) | | 0.25 (0.03-2.62) | 2.58 (0.21-7.44) | P < 0.001 | -4.197 |
| fT4, ng/dL, median(IQR) | | 1.34 (1.12-1.61) | 1.01 (0.90-1.28) | P < 0.001 | -3.856 |
| **Chi-square test (Table 3)** | | | | | |
| Variables | | Clinically non-relevant (n = 42) | Clinically relevant (n = 29) | P-value | **X^2^-value*** |
| Male, n(%) | | 8 (19) | 5 (17) | 0.847 | 0.037 |
| Reason for LT4, n(%) | PTC | 23 (54) | 23 (79) | 0.04 | 4.532 |
|  | Others | 19 (45) | 6 (20) |  |  |
| Reason for RIF, n(%) | TB | 31 (73) | 18 (62) | 0.503 | 1.374 |
|  | LTBI | 2 (4) | 3 (10) |  |  |
|  | NTM | 9 (21) | 8 (27) |  |  |
| Underlying liver disease, n(%) | | 8 (19) | 6 (20) | 0.864 | 0.029 |
| Underlying kidney disease, n(%) | | 3 (7) | 2 (6) | 1.000 | 0.002 |
| **Mann-Whiney U test (Table 3)** | | | | | |
| Variables | | Clinically non-relevant (n = 42) | Clinically relevant (n = 29) | P-value | **U-value*** |
| Age, median(IQR) | | 54 (45-60) | 49 (46-65) | 0.497 | 551.00 |
| LT4 dose, μg/kg/day, mean ± SD | | 2.20 ± 1.12 | 2.04 ± 0.63 | 0.434 | 564.50 |
| RIF dose, mg/kg/week, median(IQR) | | 69 (55-74) | 70 (45-77) | 0.874 | 595.50 |
| Time after starting RIF, weeks,  median (IQR) | | 26 (15-38) | 41 (23-57) | **0.005** | 369.00 |
| Baseline TSH level, mIU/L,  median(IQR) | | 0.21 (0.10-4.62) | 0.29 (0.08-2.40) | 0.906 | 599.00 |
| Baseline fT4 level, ng/dL, mean± SD | | 1.30 ± 0.09 | 1.42 ± 0.06 | 0.349 | 196.00 |
| **Binary logistic regression analysis with backward LRT (Table 4)** | | | | | |
| Model | | Variables | OR (95% CI) | P-value | **β-value*** |
| Model 1 | | No remnant thyroid | 3.52 (1.21-10.26) | 0.021 | 1.260 |
|  |  | Time after starting RIF | 1.03 (1.00-1.06) | 0.034 | 0.032 |
| Model 2 | | No remnant thyroid | 9.20 (2.28-37.17) | 0.002 | 2.220 |
|  |  | Time after starting RIF | 1.04 (1.00-1.08) | 0.019 | 0.042 |
|  |  | LT4 dose | 0.36 (0.16-0.79) | 0.011 | -1.010 |

*Z-value for Wilcoxon matched paired test, X^2^-value for Chi-square test, U-value for Mann-Whiney U test, and β-value for Binary logistic regression analysis with backward LRT are presented.
